# Predictors of Stress-Delta High-Sensitivity Troponin T in Emergency Department Patients Undergoing Stress Testing

**DOI:** 10.7759/cureus.29601

**Published:** 2022-09-26

**Authors:** Emily J White, Stephen J Susman, Andrew Bouffler, J. Clancy Leahy, S. Michelle Griffin, Robert Christenson, L. Kristin Newby, Alexander Gordee, Maragatha Kuchibhatla, Alexander T Limkakeng

**Affiliations:** 1 Department of Emergency Medicine, Duke University School of Medicine, Durham, USA; 2 Department of Emergency Medicine, Indiana University School of Medicine, Indianapolis, USA; 3 Department of Pathology, University of Maryland, Baltimore, USA; 4 Department of Medicine, Duke University School of Medicine, Durham, USA; 5 Department of Biostatistics and Bioinformatics, Duke University School of Medicine, Durham, USA

**Keywords:** acs risk stratification, stress test, biomarkers, high-sensitivity troponin t, myocardial ischemia

## Abstract

Background and objective

Elevations in high-sensitivity troponin T (hs-TnT) are frequently observed following extreme physical exercise. In light of this, we sought to determine whether specific clinical characteristics are associated with this phenomenon in patients undergoing cardiac exercise tolerance testing (ETT).

Methods

We conducted a retrospective analysis of a prospectively collected biospecimen repository of 257 patients undergoing a stress echocardiogram for possible acute coronary syndrome (ACS). Ischemic electrocardiogram (ECG) changes during ETT and the presence or absence of ischemia on imaging were determined by a board-licensed cardiologist. N-terminal pro-brain natriuretic peptide (NT-proBNP) and hs-TnT assays were obtained immediately before and two hours following ETT. We developed linear regression models including several clinical characteristics to predict two-hour stress-delta hs-TnT. Variable selection was performed using the least absolute shrinkage and selection operator (LASSO).

Results

The mean age of the patients was 52 years [standard deviation (SD): 11.4]; 125 (48.6%) of them were men, and 88 (34.2%) were African-American. Twenty-two patients (8.6%) had ischemia evident on echocardiography, and 31 (12.1%) had ischemic ECG changes during exercise. The mean baseline hs-TnT was 5.6 ng/L (SD: 6.4) and the mean two-hour hs-TnT was 7.1 ng/L (SD: 10.2). Age and ischemic ECG changes were associated with two-hour stress-delta hs-TnT values.

Conclusions

Based on our findings, ischemic changes in stress ECG and age were associated with an increase in hs-TnT levels following exercise during a stress echo.

## Introduction

Cardiac troponin (cTn) is a blood biomarker that is associated with several pathologic states [1]. One notable exception with unclear implications is that troponin can be elevated in presumably healthy people following physical exercise [2-7]. The mechanism of cTn release following physical exercise is postulated to be due to the increased membrane permeability of cardiomyocytes allowing unbound cTn to diffuse from the cytosol across a concentration gradient from intracellular to extracellular environments. However, the exact mechanisms and contributing clinical factors associated with the phenomenon have still not been identified.

In a meta-analysis of 1120 cases from 26 trials, Shave et al. found an elevation in post-exercise cTn in approximately half of the study participants [7]. In a systematic review by Regwan et al. that investigated the association of cTn elevation with marathon running, 0.6% of the participants had a detectable cTn before the race compared to 62% of the participants after the race [8]. Unsurprisingly, cardiac biomarker detection is even greater when a high-sensitivity troponin assay is used [9]. Although troponin elevation has been noted across a range of exercise activities, most of the attention has been focused on participants in extreme or prolonged endurance activities. It remains uncertain whether elevations can be reliably observed over shorter or less intense forms of exercise and whether it is a universal phenomenon or one observed only in particular individuals.

Given these unknowns, we sought to determine the incidence and degree of increase in high-sensitivity troponin T (hs-TnT) levels following an exercise tolerance test (ETT) and the clinical variables associated with any observed changes.

ETT is valuable for diagnosing several cardiac diseases, including heart failure with preserved or reduced ejection fraction, cardiomyopathies, pulmonary arterial hypertension, valvular heart disease, and coronary artery disease [10]. It can be performed on a treadmill or stationary exercise bicycle and data on ventilation and respiratory gas exchange are collected with a face mask or mouthpiece. The protocol typically increases the work rate gradually in a ramp-like approach in order to identify the level of effort at which the patient becomes symptomatic. ETT is frequently paired with echocardiography to combine functional and structural data. A stress echo may identify wall motion abnormalities consistent with coronary artery disease.

Patients undergoing exercise cardiac stress testing represent a different population from prior studies­ that examined cardiac troponin changes following exercise. These patients differ in terms of the duration and intensity of exercise and they have a different demographic profile. We studied several variables to determine their association with stress-test-induced changes in hs-TnT levels at two hours post-exercise.

## Materials and methods

Participants

We conducted a retrospective study of data from a prospectively collected biospecimen repository. We had previously reported the methods for the collection and analysis of samples and data [11], which we briefly outline here as follows. Patients were eligible if they were placed in an academic hospital emergency department (ED) observation unit for evaluation of potential ischemic symptoms at presentation and scheduled to receive a stress echocardiogram as part of their usual care. Patients were eligible after non-diagnostic serial electrocardiogram (ECG) and cardiac troponin tests [manufacturer’s cutoff of <0.10 mcg/mL, 10% coefficient of variance (CV) concentration = <0.03 mcg/mL] had excluded acute myocardial infarction or unstable angina. As described previously, we enrolled 383 patients over the age of 30 years in the ED observation unit for suspected acute coronary syndrome (ACS) from November 2012 to September 2014 [11]. The study, approved by the Duke Institutional Review Board (IRB), excluded patients if they were unable to give informed consent or had any narrow complex tachyarrhythmia, ventricular arrhythmias, unstable vital signs such as persistent hypotension or tachycardia, aortic aneurysm, aortic dissection or severe stenosis, pulmonary embolism, active myocarditis or pericarditis, or if they were scheduled for a stress test other than an exercise echocardiogram. The stress test required all beta-blocking medications to be stopped at least 24 hours beforehand, and according to the Bruce Protocol, we halted the procedure if patients had symptoms of angina, >/=3 mm ST-segment depression, a >/= 20 mmHg drop in systolic blood pressure (BP), ventricular arrhythmias, any narrow complex tachyarrhythmia with a rapid ventricular rate, fatigue, or severe dyspnea.

Stress testing

For the determination of ischemia on imaging, we performed an echocardiogram immediately before and after the stress test. A board-certified cardiologist interpreted all tests for inducible ischemia based on new segmental wall motion abnormalities upon exercise as part of usual care. For the study data, two additional investigators independently reviewed the imaging reports, with a third independent investigator, a board-certified cardiologist with access to the patient's clinical records and stress test report, resolving any disagreements. Each investigator examining the exercise echocardiogram report, as well as the performing cardiologist, was blinded to the results of the biomarker blood tests at the time of their decisions. In a similar fashion to the echocardiogram interpretations, a board-certified cardiologist examined the ECG tracing recorded during the stress test, and categorized findings as either “ischemic”, “non-ischemic”, or “non-diagnostic/equivocal” in their report as part of usual care. All stress ECG reports labeled as “ischemic” had at least 1 millimeter of ST segment depression in at least two or more contiguous leads. We collected the demographics, including age, sex, race, and the presence or absence of diabetes, hypertension, and smoking.

Blood sample collection and analysis

ED staff collected blood samples for research purposes via a peripheral blood draw, usually through an existing intravenous (IV) catheter. We took samples less than one hour before the stress test, and two hours, and four hours after. We centrifuged the blood within an hour of collection, with plasma measured in 0.5-milliliter aliquots. Samples were then frozen at -80 °C and later run at the University of Maryland using the Highly Sensitive Troponin T assay from Roche Diagnostics (Indianapolis, IN). The Limit of Blank for this assay is 3.0 nanograms per liter (ng/L), the Limit of Detection is 5.0 ng/L, and the manufacturer’s 99th percentile cutoff has been established as 14 ng/L. At a level of 27.6 ng/L, the CV for the system is 3.6%; at a level of 2,110.5 ng/L, the CV for this assay is 2.6%. As per the laboratory protocol, we counted measurements below 3.0 ng/L as 1.5 ng/L. One patient had a two-hour stress-delta hs-TnT value of 113.5, which was deemed implausible. For analyses, we used the Winsorized mean, replacing this value with the next highest value of 15. The N-terminal pro-brain natriuretic peptide (NT-proBNP) detection limit was 5.0 ng/L; we counted measurements below this limit as 2.5 ng/L. The 97.5th percentile cutoff has been established as 115 ng/L, and the manufacturer-recommended cutoff for clinical use is 125 ng/L. At a level of 125 ng/L, the CV for the system is 2.7%. We excluded patients from the study if they had missing samples, mild to moderate hemolysis (>10 mg/dL), or if there were technical difficulties in the assay that prevented a reliable measurement. We blinded both laboratories to the corresponding clinical information of each sample to eliminate bias. We collected and processed the data using the REDCap (Research Electronic Data Capture) data capture tool hosted at Duke University, Durham, NC.

Outcome determination

The primary outcome was two-hour hs-TnT stress-delta values. We also determined variables associated with changes in hs-TnT levels following exercise stress testing via linear regression. Potential predictor variables included age, sex, race, ischemia on imaging, baseline hs-TnT, baseline NT-proBNP, diabetes, hypertension, smoking, or ischemic changes in ECG tracings during stress. We chose these factors due to their known associations with the presence or absence of cardiovascular disease, which could affect levels of hs-TnT. Analyses were performed using R version 3.6.3 (R Foundation for Statistical Computing, Vienne, Austria, 2019). The least absolute shrinkage and selection operator (LASSO) was utilized in order to select a suitable subset of covariates that were predictive of changes in the two-hour stress-delta hs-TnT. Additionally, a backward selection method utilizing Akaike’s Information Criterion (AIC) to select a subset of variables was used.

## Results

Descriptive statistics

In total, 383 patients with suspected ACS being evaluated with exercise stress echocardiogram tests were enrolled. Of them, 123 were excluded for missing measurements of either the baseline or two-hour post-stress test hs-TnT. Two additional patients were excluded for indeterminate stress-test imaging results and one patient was excluded for missing medical history, leaving 257 patients in the analysis dataset.

Table 1 lists the patient demographics and clinical characteristics. The mean baseline hs-TnT concentration was 5.6 ng/L [standard deviation (SD): 6.4] and the mean two-hour hs-TnT value was 7.1 ng/L (SD: 10.2); the mean individual stress-delta was 1.1 ng/L (SD: 2.8) (Table 1). Twenty-two patients (8.6%) had ischemia on echocardiography. Thirty-one patients (12.1%) had ischemic changes on the exercise stress ECG, and 207 (80.5%) had non-ischemic ECG results. Four patients (1.6%) had equivocal exercise stress ECG results, and 13 (5.1%) had non-diagnostic results, which were combined for the purpose of calculations.

**Table 1:** Patient demographics and clinical characteristics SD: standard deviation; IQR: interquartile range; ECG: electrocardiogram; hs-TnT: high-sensitivity troponin T; NT-proBNP: N-terminal pro-brain natriuretic peptide

Characteristic	Values (n=257)
Age in years	
Mean (SD)	52.2 (11.4)
Median (IQR)	51 (44 - 59)
Min - Max	30 - 89
Male gender, n (%)	125 (48.6%)
Race, n (%)	
Asian	2 (0.8%)
Black	88 (34.2%)
Native Hawaiian or Pacific Islander	0 (0.0%)
White	161 (62.6%)
Other	6 (2.3%)
Diabetes, n (%)	44 (17.1%)
Hypertension, n (%)	121 (47.1%)
Current smoker, n (%)	150 (58.4%)
Hyperlipidemia, n (%)	89 (34.6%)
Cocaine use, n (%)	
Current	3 (1.2%)
Past (none for the past month)	17 (6.6%)
Never	233 (90.7%)
Coronary artery disease, n (%)	14 (5.4%)
Imaging results, n (%)	
Ischemia	22 (8.6%)
No ischemia	235 (91.4%)
Exercise stress ECG, n (%)	
Equivocal	4 (1.6%)
Missing	2 (0.8%)
Non-diagnostic	13 (5.1%)
Non-ischemic	207 (80.5%)
Ischemic	31 (12.1%)
Baseline hs-TnT	
Mean (SD)	5.6 (6.4)
Median (IQR)	4.1 (1.5 - 7)
Min - Max	1.5 - 65.3
2-hour hs-TnT	
Mean (SD)	7.1 (10.2)
Median (IQR)	5 (1.5 - 8.1)
Min - Max	1.5 - 122.7
4-hour hs-TnT	
Mean (SD)	9.2 (10.6)
Median (IQR)	6.1 (2.9 - 10.5)
Min - Max	1.5 - 74.2
Missing	134 (52.14%)
Baseline NT-proBNP	
Mean (SD)	81.3 (184.8)
Median (IQR)	33.3 (10 - 77.9)
Min - Max	2.5 - 2342.5
Missing	1 (0.39%)
2-hour delta hs-TnT	
Mean (SD)	1.1 (2.8)
Median (IQR)	0.4 (0 - 2.2)
Min - Max	-6.7 - 14.9

**Figure 1:**
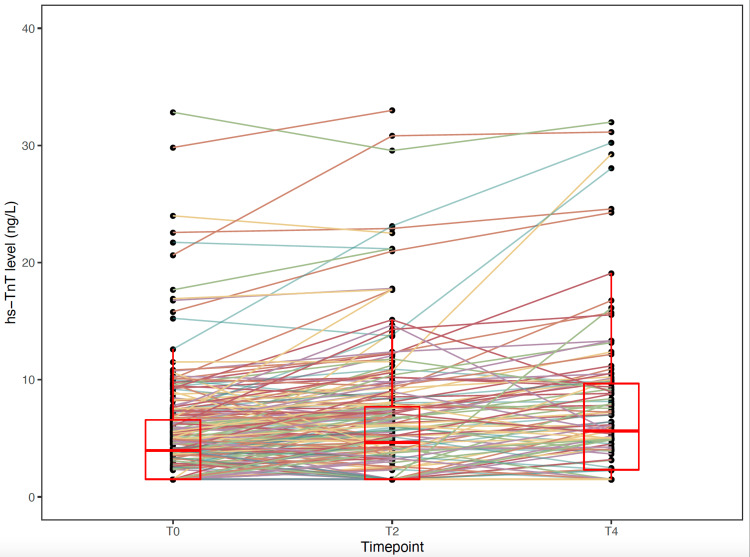
Delta troponins in the non-ischemic group hs-TnT: high-sensitivity troponin T

**Figure 2:**
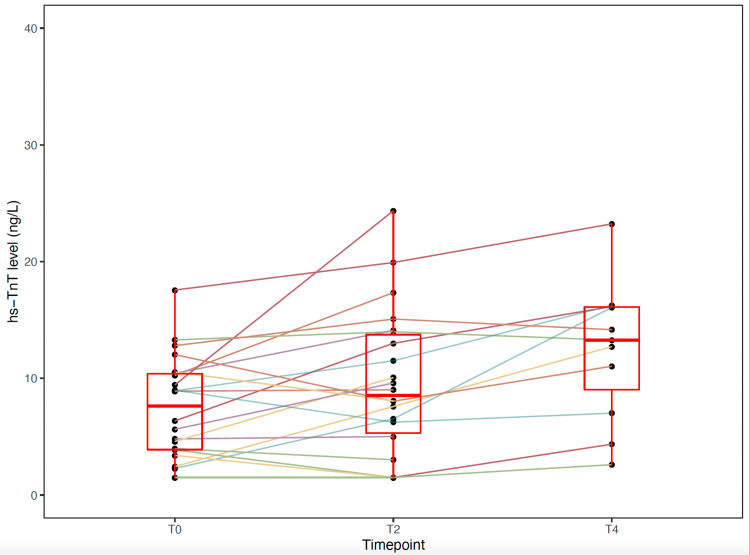
Delta troponins in the ischemic group hs-TnT: high-sensitivity troponin T

In Figure 1 and Figure 2, delta troponin levels are visualized for non-ischemic and ischemic groups.

Table 2 demonstrates the results of the baseline troponin levels drawn immediately before the exercise stress test, as well as those drawn at two hours and four hours post-stress test.

**Table 2:** Troponin levels at baseline, two hours, and four hours hs-TnT: high-sensitivity troponin T

hs-TnT at T0		hs-TnT at T2			hs-TnT at T4			
		<3 ng/L	3 - 14 ng/L	>14 ng/L	<3 ng/L	3 - 14 ng/L	> 14 ng/L	Missing
<3 ng/L	102 (39.8%)	73 (71.6%)	28 (27.5%)	1 (0.1%)	24 (23.5%)	20 (19.6%)	3 (2.9%)	55 (53.9%)
3 - 14 ng/L	140 (54.7%)	13 (9.3%)	117 (83.6%)	10 (7.14%)	7 (5.0%)	52 (37.1%)	10 (7.14%)	71 (50.7%)
>14 ng/L	14 (5.5%)	0 (0.0%)	1 (7.1%)	13 (92.9.%)	0 (0.0%)	0 (0.0%)	7 (50.0%)	7 (50.0%)

Linear regression

In fully unadjusted linear regression models (Table 3), age and ischemic changes in stress ECG were the only covariates that were significantly associated with stress-delta hs-TnT. Without adjusting for other variables, each additional year of a patient’s age was associated with an increase of 0.05 ng/L [95% confidence interval (CI): 0.02, 0.08] in a patient’s two-hour stress-delta hs-TnT, and the proportion of variation in the two-hour stress-delta hs-TnT explained by age alone was 4%. Ischemic changes in stress ECG were associated with an increase of 2.13 ng/L (95% CI: 1.09, 3.17) in the two-hour stress-delta hs-TnT, and a non-diagnostic/equivocal stress ECG result was associated with a decrease of -0.30 (95% CI: -1.60, 0.99). The proportion of variation in the two-hour stress-delta hs-TnT explained by the ECG results alone was 6%.

**Table 3:** Unadjusted coefficient estimates NT-proBNP: N-terminal pro-brain natriuretic peptide; hs-TnT: high-sensitivity troponin T; ECG: electrocardiogram

Predictor	Estimate (95% confidence interval)	P-value	R-squared
Age	0.05 (0.02, 0.08)	0.001	0.04
Gender (female)	-0.39 (-1.09, 0.3)	0.266	<0.01
Race (black)	-0.49 (-1.23, 0.25)	0.192	<0.01
Race (other)	-0.25 (-2.27, 1.76)	0.804	
Diabetes	0.00 (-0.92, 0.92)	0.996	<0.01
Hypertension	0.24 (-0.46, 0.93)	0.505	<0.01
Smoking	-0.24 (-0.94, 0.47)	0.507	<0.01
Hyperlipidemia	0.32 (-0.41, 1.05)	0.393	<0.01
Cocaine (current or past)	-0.29 (-1.59, 1.01)	0.661	<0.01
Coronary artery disease	-0.4 (-1.93, 1.13)	0.608	<0.01
Baseline NT-proBNP	0.00 (0.00, 0.00)	0.689	<0.01
Baseline hs-TnT	0.05 (0, 0.11)	0.060	0.01
Imaging results (ischemia)	1.00 (-0.23, 2.24)	0.111	<0.01
Ischemic ECG	2.13 (1.09, 3.17)	<0.001	0.06
Non-diagnostic ECG	-0.30 (-1.6, 0.99)	0.646	

LASSO

LASSO selected age and ischemic ECG changes during stress testing, consistent with the findings from the univariate models. Additionally, a backward selection method utilizing AIC to select a subset of variables was used, and the results were consistent with the previously described LASSO analyses. The unpenalized results of this model are shown in Table 4, along with 95% CIs and p-values corrected for the use of the LASSO in variable selection [12].

**Table 4:** Unpenalized coefficients from the LASSO model with corrected 95% confidence intervals and p-values LASSO: least absolute shrinkage and selection operator; ECG: electrocardiogram

Variable	Estimate (95% confidence interval)	P-value
Age	0.04 (0.01, 0.07)	0.003
Ischemic ECG	2.03 (0.99, 3.06)	<0.001

A sensitivity analysis was performed in which the original implausible two-hour stress-delta hs-TnT value of 113.5 was included. The results from both the LASSO and backward selection methods were unchanged. Furthermore, the inclusion of stress ECG results that were labeled as non-diagnostic or equivocal resulted in similar findings.

## Discussion

In the setting of suspected ACS, cardiac troponin is the most commonly used biomarker to quantify the risk of cardiac ischemia or injury. It can be elevated in a variety of other scenarios as well, including after extreme exercise in presumably healthy athletes. A number of factors are thought to influence the degree to which troponin elevation following exercise can be observed. In this study, we examined variables associated with hs-TnT elevation following the brief, graded exercise associated with a cardiac stress test in recently symptomatic ED patients. Our study adds to our understanding of factors that influence troponin release in the setting of exercise by studying patients who were older, had brief exercise activity, and had an objective assessment of the presence or absence of ischemia by both ECG and echocardiographic imaging. The results of this study show that age and ischemic ECG changes during exercise are weakly associated with an increase in hs-TnT values from pre- to post-stress test but account for a small proportion of the variation seen.

The causes of exercise-induced troponin elevation are unclear. The prevailing explanation is that exercise induces increased membrane permeability in cardiomyocytes allowing unbound cytosolic troponin to diffuse into the extracellular environment. Alternatively, it has been suggested that mechanical stress through the transient disruption of the sarcolemma is responsible for this increased membrane permeability. This has been seen in the skeletal muscle of rats after downhill running in which the cell wounds resolved within 24 hours, correlating with the time course of the detection of creatine kinase levels [13]. It has also been demonstrated in-vivo cardiac muscle and was associated with the release of growth factors [14,15].

Generally, troponin elevations have been studied in the setting of extreme exercise. Exercise-induced cTn release is apparent in almost half of the endurance athletes studied [7,8]. A systematic review that included 10 studies investigating hs-TnT elevation after running found that all participants demonstrated a detectable level of hsTnT following completion of the race and that 69.8% had a value above the 99th percentile, the cutoff used for myocardial necrosis [9]. In those studies, the duration and intensity of exercise appear to be key predictors of troponin elevation [16,17]. Since we were using data from a repository collected for a different purpose, we did not systematically record the duration or intensity of the patient’s exercise activity. However, patients followed a standardized protocol, and hence exercise duration ranged from 1 to 20 minutes. Even at the extreme end, this is significantly less time than that reported for prior studies of endurance athletes, and there was also less variation in intensity. We found relatively low rates of troponin elevation during these brief exercise periods.

Our finding that age is a predictor of troponin elevation differs from existing literature on endurance events [7,8]. Prior literature has suggested that the phenomenon is not dependent on age and appears to occur even in children and adolescents undergoing intense exercise [17,18]. However, our study population differed in a few notable ways. In this study, we studied an older patient population. The average age of the participants in studies included in systematic reviews of exercise-related troponin elevation ranged from 21 to 44 years [7] and 29 to 49 years [8], compared with our study’s median age of 51 years. A prior study of older patients engaging in endurance events showed comparable rates of troponin elevation to younger patients [19]. Thus, prior training and conditioning may be more important factors, as well as comorbidities associated with undiagnosed coronary artery disease that are present in our study population. This may ameliorate exercise-induced elevations in troponin [20,21]. Although we did not have systematic data on the degree of physical conditioning our patients had, it is highly likely that they had less training than participants in endurance events.

Other studies on patients undergoing stress testing confirm that brief exercise seen in a stress test is generally not associated with significant changes in hs-TnT, even among patients with obstructive coronary disease [22,23]. A prior analysis of participants in this study [11] noted that there is no relationship between delta hs-TnT values and the presence or absence of ischemia on stress echocardiography. In this study, however, we identified that ischemic changes in stress ECG were more predictive of the variation in hs-TNT during a stress test compared with stress imaging. The reasons for this are unclear. It is possible that electrocardiographic changes identify patients with increased cardiomyocyte membrane permeability, whereas ischemic imaging reflects difficulties in contractility.

Limitations

This study is limited in that the study population was selected from a single hospital system and patients were recently symptomatic of possible ACS. Thus, despite the fact that most patients had normal stress test imaging results, our patients would not be considered a completely “healthy” patient population. Additionally, most patients were at low risk for coronary artery disease, and median baseline values of hs-TnT were very low. The interquartile range for stress delta hs-TnT was 0-2.2 ng/L, indicating that the majority of patients did not have substantial changes in hs-TnT after stress testing. This limited amount of variation may limit our ability to detect subpopulations that are more likely to demonstrate changes.

## Conclusions

Based on our findings, ischemic changes in stress ECG and age were associated with an increase in hs-TnT levels following brief exercise during a stress test. The proportion of variation explained by each of these factors alone was small. Several other factors studied were found to have no statistically significant association. Future studies should examine whether there are important differences in biomarkers in patients with electrocardiographic compared to echocardiographic inducible ischemia.
